# Is the Deterioration of Children’s Mental Health a Price We Pay for the COVID-19 Pandemic?

**DOI:** 10.3389/ijph.2023.1606744

**Published:** 2023-11-07

**Authors:** Andrea Madarasova Geckova

**Affiliations:** Department of Health Psychology and Research Methodology, University of Pavol Jozef Šafárik, Košice, Slovakia

**Keywords:** mental health, COVID-19, pandemic, health behavior, school-age children

The first post-pandemic findings on adolescents’ mental health and wellbeing from the Health Behavior in School-aged Children (HBSC) survey are alarming [1]. Older girls seem to be affected the most, as two-thirds of 15 years old girls reported experiencing multiple health complaints, and nearly one-third had felt lonely most of the time or always in the past year [1]. The decline of life satisfaction and self-reported health between the HBSC survey in 2017/2018 and 2021/2022 was evident across most countries [1]. Is the deterioration of children’s mental health a price we pay for the COVID-19 pandemic?

Being an adolescent in the times of COVID-19 entailed much more than the risk of the disease itself. Various restrictions in their lives resulting from pandemic management, public and private discourses over it inciting negative feelings, school containment policies, changes in parents’ working conditions, and changes in their daily routines might have a varied impact on various communities. The study of Jansen et al. [2] offers an overview of COVID-19 childcare and school containment policies in 19 European countries. There was a huge difference between countries in the number of measures applied, the degree of obligation, and the duration for which they were applied. At the same time, the effectiveness of the measures, as well as their unwanted detrimental effect on the mental health of children, were rarely reported, so we do not know if the application of these measures was worth it.

The sense of unity, or in other words positive feelings about being a part of a larger social structure, was associated with better health outcomes in Swedish adolescents (*n* = 1,392, 15 years old). However, adolescents with a lower socioeconomic position, an immigrant background, or perceiving lower social support from family, classmates, and teachers reported a lower sense of unity [3]. We might hypothesize that being an adolescent in the times of COVID-19 might strengthen or weaken the sense of unity in particular communities, based on their experience with pandemic management. Do the adolescents feel cared for or left behind during the pandemic? Do they feel a part of a society battling the pandemic or do they feel excluded?

A longitudinal Swiss study (*n* = 2,534, 5–16 years old, June 2020/April 2021) indicates the detrimental impact of the COVID-19 pandemic on children’s physical activity, screen time, and sleep duration. However, it also shows that children who were able to follow the recommendations for all three types of behavior during this period were more likely to report excellent health and higher life satisfaction than children not meeting any recommendations [4]. Who are the children who have been able to maintain a healthy lifestyle despite all the restrictions? And could we identify children at risk and mitigate that risk in the future?

Very inspiring are the findings of a Canadian study revealing no notable changes in mental health and wellbeing pre-pandemic vs. post-lockdown among school-aged children (*n* = 476, 9–12 years old) from schools in socioeconomically disadvantaged communities participating in a school-based health promotion program targeting healthy lifestyle behaviors and mental wellbeing [5]. It appears that implemented health promotion programs are mitigating the impact of the pandemic on children’s mental health and wellbeing. Moreover, we learned that interventions which are co-developed with adolescents are more effective than those in which they only participate [6]. Have we learned this lesson? Are our schools better prepared, do they have enough competencies and capacity to deliver such programs?

The measures to contain the COVID-19 pandemic also had an impact on parents lives [7]. During the lockdown period, in 30% of the families, at least one parent had to work remotely, and 32% of families reported that at least one parent experienced a reduction or loss of income during the lockdown. Children from these families had a lower health-related quality of life in June–July 2020 compared to children from families in which both parents maintained regular working conditions, and this remained the case in January and March 2021 [7]. Changes in parents’ working conditions are one of the possible adverse events that children might have experienced during the COVID-19 pandemic. Recent studies on Slovak adolescents [8, 9] show that the accumulation of adverse events is associated with emotional and behavioral problems in adolescents, while resilience and feeling hopeless might mediate this association. Being an adolescent in the time of COVID-19 might add the final drop to an already existing load of burdens, resulting in a collapse of resilience resources.

Based on my knowledge, we do not yet know the full extent of the impact of the COVID-19 pandemic on children’s mental health, but there are bodies of knowledge that suggest we need to act now, not only to rebuild the resilience of the younger generation, but also to develop sufficient preparedness of our communities to promote the resilience of the younger generation [10]. The pandemic of COVID-19 was just one of many challenges that this generation is facing and will face in the future.
